# 911. Rapid Hepatitis C Treatment Initiation in Young People Who Inject Drugs: Final Results from the HCV-Seek, Test and Rapid Treatment (HCV-ST&RT) Randomized Pilot Clinical Trial

**DOI:** 10.1093/ofid/ofab466.1106

**Published:** 2021-12-04

**Authors:** Benjamin Eckhardt, Shashi Kapadia, Melinda Smith, Yesenia Aponte-Melendez, Chunki Fong, La Davis, Pedro Mateu-Gelabert, Kristen Marks, Kristen Marks

**Affiliations:** 1 NYU School of Medicine/Bellevue Hospital, New York, NY; 2 Weill Cornell Medicine, New York, NY; 3 CUNY School of Public Health, New York, New York

## Abstract

**Background:**

Young people who inject drugs (PWID) have higher HCV incidence and lower treatment initiation rates compared to their older peers. Novel, simplified care models need to be developed to engage, treat and cure hard to reach patient populations, such as young PWID.

**Methods:**

We present final data from the randomized pilot clinical trial *HCV-Seek Test & Rapid Treatment* (HCV-ST&RT) for curing HCV in young PWID. Eligible participants were 18-29 years of age, HCV Ab+, treatment naïve, and had injected drugs in the past 30 days. Participants were randomized 1:1 to the Rapid Treatment or Usual Care arm. Participants randomized to ***Rapid Treatment*** received a same-day medical evaluation, confirmatory and baseline lab testing, and 7-day starter pack of sofosbuvir/velpatasvir. Participants in ***Usual Care*** received same-day HCV confirmatory testing and, if positive, facilitated referral to local providers. The primary endpoint was sustained virologic response (SVR12) in RNA+ participant within 12 months of enrollment.

**Results:**

Among 47 eligible participants, 38 were enrolled and randomized. 14/18 in the Rapid Treatment arm and 11/20 in the Usual Care arm were confirmed HCV RNA+ and included in the intention-to-treat analysis. Demographics of the RNA+ participants were similar in the two arms with a mean age of 26 years; 24% women; 36% Hispanic and 4% non-Hispanic black. At baseline 24% were homeless, 52% received medication for opioid use disorder in the prior 90 days, and participants injected a median of 20 of the last 30 days. In the intention-to-treat analysis, 9/14 (64%) of the Rapid Treatment arm and 2/11 (18%) of the Usual Care arm had confirmed SVR12 (p=0.042). Of the 5 participants in the Rapid Treatment arm who did not achieve SVR12, 1 had treatment failure, 1 never started DAA therapy, 1 had an on-treatment response with pending SVR12 confirmation, and 2 were lost to follow-up.

**Conclusion:**

Among young HCV RNA+ PWID, significantly higher rates of cure were achieved using the Rapid Treatment model with same day, low-threshold, simplified HCV care compared to facilitated referral. Meeting young PWID where they’re at and initiating HCV treatment ‘in the moment’ without the need for repeat visits appears to be a promising strategy for treating this hard to reach population.

**Disclosures:**

**Benjamin Eckhardt, MD, MS**, **Gilead Sciences** (Grant/Research Support) **Shashi Kapadia, MD**, **Gilead Sciences Inc** (Grant/Research Support) **Kristen Marks, MD**, **Gilead Sciences** (Grant/Research Support)

